# Adverse childhood experiences: impacts on adult mental health and social withdrawal

**DOI:** 10.3389/fpubh.2023.1277766

**Published:** 2023-10-26

**Authors:** Manabu Wakuta, Tomoko Nishimura, Yuko Osuka, Nobuaki Tsukui, Michio Takahashi, Masaki Adachi, Toshiaki Suwa, Taiichi Katayama

**Affiliations:** ^1^Research Department, Institute of Child Developmental Science Research, Hamamatsu, Japan; ^2^United Graduate School of Child Development, Osaka University, Suita, Japan; ^3^Department of Neuropsychiatry, Graduate School of Medicine, Hirosaki University, Hirosaki, Japan; ^4^Research Center for Child Mental Development, Hamamatsu University School of Medicine, Hamamatsu, Japan; ^5^Smart-Aging Research Center, Tohoku University, Sendai, Japan; ^6^Department of Psychology, Meiji Gakuin University, Yokohama, Japan; ^7^Department of Health and Welfare, Kawasaki University of Medical Welfare, Okayama, Japan

**Keywords:** adverse childhood experiences, ACEs, school, social withdrawal, Hikikomori, bullying

## Abstract

**Background:**

Adverse childhood experiences (ACEs) have been found to negatively impact adult mental health outcomes. Numerous studies have highlighted on ACEs in family and community settings. However, few have examined the impact of ACEs in school settings, despite the potential influence on social participation. Hikikomori, characterized by severe social withdrawal, was first studied in Japan and has gained recognition in recent years. The present study aims to present the concept of ACEs specific to schools and investigate the impact of both school ACEs and traditional ACEs on adult mental health and Hikikomori.

**Methods:**

A total of 4,000 Japanese adults, aged 20–34, were recruited through an Internet survey form. All data were obtained in October 2021. Participants answered questions regarding their ACEs in the family (10 items), school ACEs (five teacher-related items and two bullying-related items), depressive/anxiety symptoms, and Hikikomori (remaining at home for more than 6 months).

**Results:**

A significant association with depressive/anxiety symptoms was shown in both ACEs and school ACEs. An increase of one point in the ACE scores was associated with a 24% increase in the risk of depressive/anxiety symptoms. School ACE scores also demonstrated a significant association with depressive/anxiety symptoms, with an increase of one point associated with a 44% increase in the risk of these symptoms. As for Hikikomori, a significant association was shown in the school ACEs only: a 29% increased risk of Hikikomori for every one-point increase in school ACE scores. Both school ACE scores for teacher-related and bullying-related factors revealed a significant association with Hikikomori; the rates of increased risk were 23 and 37%, respectively.

**Conclusion:**

These results suggest that school ACEs, rather than ACEs in the family, are associated with the risk of Hikikomori. School ACEs are important for social adaptation, and reducing traumatic experiences in school settings may have the potential to prevent problems in later life, specifically in terms of social participation.

## 1. Introduction

### 1.1. Adverse childhood experiences studies

ACEs encompass highly distressing events that children may experience, such as child abuse, domestic violence, and parental substance abuse (1). These ACEs are associated with illicit drug use, mental illness, and cardiovascular diseases (1). The economic burden of ACEs is substantial, estimated to be $581 billion in Europe and $748 billion in North America, with 75% of the cost incurred by individuals who have experienced two or more ACEs (1).

The ACEs study conducted by Kaiser Permanente and the Centers for Disease Control and Prevention in the United States is a representative research (2). This study found that ACEs have enduring effects on both physical and social aspects of wellbeing throughout one's life (3). Subsequent research has corroborated these findings, highlighting the impact of ACEs on health outcomes during adulthood (4–6). Thus, the prevention of ACEs has significant implications not only for personal wellbeing, but also for social stability and reduction of financial burdens. The association between ACE scores and health and social problems in adulthood is usually proportional, particularly when ACE scores are ≥4, and the risk increases dramatically (7).

The prevalence of ACEs is known to differ based on socio-economic status (8), and race (9), and changes over time (10). However, it is likely that the impact of ACEs transcends national and cultural differences (11). Consequently, the ACEs study has spread worldwide and has been conducted in various regions, including Asia. For instance, Qu et al. (12) reported that at least one adverse experience had been encountered by 51.2% of elementary and junior high school students in China. Additionally, Lin et al. (13) found that 89.9% of Chinese adults aged 45 and older had at least one ACE, with 18.0% having ≥4. Similarly, Wang et al. (14) investigated ACEs among Taiwanese youth and found that 61.6% had at least one ACE. In Japan, the prevalence of experiencing at least one ACE, among adults, is ~27–40% (15–17). According to a review by Bellis et al. (1), research conducted in the USA indicated that individuals with at least one ACE accounted for 52–67%, while in Europe, this figure ranged from 25 to 53%. Notably, Japan exhibits a tendency toward lower ACE scores than other countries. Therefore, referencing the ACEs study when making policy decisions to support children and develop social systems would be beneficial.

### 1.2. Expanded ACEs

The ACEs study initially focused on estimating the prevalence and examining the association with outcomes, but has since expanded in various directions. One key aspect of the discussion is regarding the expansion of the ACEs concept itself. The original ACE scale only encompasses experiences of physical and emotional abuse within the family; it does not include other types of ACEs such as experiences of discrimination in the community, or harm inflicted by friends outside the home. Consequently, the criteria for expanding ACEs were established by the World Health Organization (WHO), including biological relevance (i.e., eliciting a biological stress reaction), policy sensitivity, prevalence across societies (neither too high nor too low), ease and speed or measurement, and proximity to causality (18, 19). Additionally, the suggestion of adding “exposure to community violence” was proposed (20).

Cronholm et al. (20) investigated additional items for the expanded ACEs, such as witnessed violence, experienced discrimination, unsafe neighborhoods, experienced bullying, and lived in foster care. Moreover, they found that these items were also associated with poverty (20). Thurston et al. (21) focused on community-level ACEs, such as community violence and racial discrimination, highlighting the need to consider ACEs at the community level. Moreover, Masuda et al. (22) found that extra-familial ACEs, such as exposure to community violence, exhibited a stronger correlation with psychosomatic symptoms compared to intra-familial ACEs, in a study of Japanese university students. SmithBattle et al. (23) reported that the most commonly discussed expanded ACE items in prior research included exposure to community violence, economic hardship in childhood, bullying, absence or death of parent or significant others, and discrimination, with the former being the most frequently mentioned.

Furthermore, recent studies utilizing the National Study of Child Health, which has been extensively discussed, have included exposure to community violence and discrimination as additional ACE items. It is widely acknowledged that the original ACE items alone are insufficient, and further research is warranted (20).

### 1.3. School ACEs

Previous research has shown that the impact of ACEs cannot be solely attributed to experiences within the household, but also extends to experiences outside of the home. Sweeting et al. revealed that the effects of bullying are equally severe to those of ACEs, based on a survey conducted on a representative sample in the United States (24). Bullying, due to its impact on physical health risks similar to other ACEs, should be categorized as a new item within the ACEs framework (25).

Instances of teachers causing harm to children and the subsequent negative effects on wellbeing have been reported. Gershoff (26) stated that although physical punishment is permitted in 35% of countries, evidence suggesting that it enhances learning is non-existent. Instead, it increases the risk of dropping out and teacher or school avoidance. Research conducted by Nearchou (27) in Greece found that experiences of psychological abuse from teachers predict problematic behavior, with a significant number of children (64%) having been victims of psychological abuse from teachers.

Moreover, Monsvold et al. (28) reported that experiencing bullying victimization by teachers is associated with an increased risk of personality disorders. Delfabbro et al. (29) also highlighted that bullying by teachers occurs as frequently as bullying by peers, with low-achieving children being more vulnerable to being victimized by teachers.

These findings suggest that various harmful experiences occur in school settings, including bullying, physical punishment, inappropriate teaching methods, and harassment, which may have long-lasting negative impacts similar to ACEs in adulthood. However, previous ACEs studies have only included witnessing or experiencing community violence as an additional item to original ACEs, with questions limited to peer bullying in school settings and no inclusion of teachers or other school staff as perpetrators (30, 31).

### 1.4. Hikikomori or social withdrawal

It is well-known that ACEs have a negative impact on employment and work performance. Individuals with ACEs are at a higher risk of joblessness and poor work performance (32, 33). One form of social withdrawal is known as Hikikomori, which was first studied in Japan and has recently gained attention worldwide (34, 35). Hikikomori is characterized by a refusal to leave one's home or room (36, 37) and is associated with suicide, compulsive behavior, and dependent behavior (38). The prevalence of Hikikomori in Japan was estimated to be 2.05% (39), 1.9% in Hong Kong (40), and 2.3% in South Korea (41). The high prevalence of Hikikomori has negative implications for society. Kato et al. (37) proposed a hypothetical model in which family factors, such as strong maternal and weak paternal relationships, and school factors, such as a less competitive environment (yutori-kyoiku) or highly competitive atmosphere (juken war), contribute to the occurrence of Hikikomori. However, no studies have quantitatively examined whether adverse experiences in school are associated with the risk of Hikikomori. Furthermore, ACEs have been reported to increase the risk of employment problems (32, 42) and workplace bullying (43), but no studies have investigated the association between ACEs and Hikikomori.

## 2. About this study

In this study, we introduce the concept of “school ACEs” in addition to the original ACEs items. We recognize that teachers in school settings may have the potential to harm children, resulting in long-lasting effects. School ACEs encompass peer bullying (classmates and upper classmen) that commonly occurs in schools. This additional item is integrated within the existing ACEs item on abuse, with the distinction being that teachers are identified as the perpetrator as opposed to family members.

Although bullying has already been examined, experiences of being hurt by teachers and other school staff are likely to satisfy the criteria for ACEs expansion set by WHO (18). In Japan, corporal punishment occurs in 0.63% of elementary schools, 1.33% of junior high schools, and 3.51% of high schools despite being prohibited by law (44). Here, corporal punishment refers to physical acts such as hitting, kicking, and shoving by teachers and does not include psychological effects such as verbal violence, threats, ignoring, or negative evaluations. According to a survey by the Tokyo Metropolitan Government (45), verbal violence occurs more often than corporal punishment, and the possibility that children may be harmed by non-corporal punishment by teachers cannot be ruled out, making the examination of school ACEs crucial (45).

This study investigates the outcomes of the school ACEs as possible worsening mental health in adulthood and social withdrawal, Hikikomori.

Mental health deterioration in adulthood must be prevented as it is a societal burden and a personal or family problem (46, 47). Mental health deterioration is a representative outcome of ACEs (1, 3, 4, 12, 48), and worsening of mental health in young people is likely associated with a wide range of individual and societal issues such as marital status and household income in adulthood (46). However, the association between school ACEs and deteriorating mental health is yet to be explored.

Regarding Hikikomori, according to the occurrence model shown by Kato et al. (37), stressful life events at school or workplace cause evasive behavior, which leads to Hikikomori. The presence of school ACEs, such as bullying and receiving reprimand by teachers, within the category of stressful life events, suggests a potential association between school ACEs and Hikikomori. However, no research has been conducted to substantiate this claim.

Therefore, this study aims to assess the prevalence of ACEs and school ACEs in Japan. Furthermore, we aim to examine the association between school ACEs and the deterioration of adult mental health, as well as their association with Hikikomori.

Our research questions are as follows:

#1 What is the prevalence of ACEs and school ACEs in Japan?
#2 How are ACEs and school ACEs related to adult depressive/anxiety symptoms?
#3 How are ACEs and school ACEs related to “Hikikomori”?

## 3. Methods

### 3.1. Participants

A total of 4,000 Japanese adults, ages 20–34, were recruited through an Internet survey form (Survey Research Center Co., Ltd. Tokyo, Japan). For comparison purposes, the ages of the participants in this study were matched to those in the Cabinet Office's Survey on Hikikomori conducted in (15–39 years of age) Japan (39). However, due to ethical considerations, the target age was set at 20 and above. In addition, because the Japanese school education system differed greatly between the 35–39 and ≤ 34 age groups, the survey was limited to ≤ 34 age group. Age groups were divided into 20–24, 25–29, and 30–34, with each group recruited to ensure equal numbers and sex ratios. A total of 1,333 participants were included in the 20–24 age group (49.3% male, 49.3% female, and 1.4% non-response), 1,334 in the 25–29 age group (49.3% male, 49.3% female, and 1.4% non-response), and 1,332 in the 30–34 age group (49.6% male, 49.7% female, and 0.7% non-response).

Initially, a trap question (“Do not answer this question”) was inserted into a survey to filter out respondents who are not answering honestly or carefully. Only those participants who passed the trap (i.e., those who did not respond to the item) were included. One participant who passed the trap but provided the same response to all questions including an invert scale was excluded from the analysis. All data were obtained in October 2021.

The survey was conducted anonymously. A written explanation of the survey was presented online, and consent for participation was obtained by checking the “I agree” box. Participants were rewarded with points (Japanese yen equivalent: 4 yen) that could be used online as compensation for their participation herein. The Hirosaki University Ethics Committee approved this study (reference No: 2021-011).

### 3.2. Measures

#### 3.2.1. ACEs

Using the ACEs Questionnaire, childhood trauma was measured (3). The questionnaire assesses 10 types of childhood trauma. Five are personal: physical abuse, verbal abuse, sexual abuse, physical neglect, and emotional neglect. Five are family dysfunctions: a parent who is an alcoholic, a mother who is a victim of domestic violence, a family member in jail, a family member diagnosed with a mental illness, and the disappearance of a parent through divorce, death, or abandonment. Responses were binary, “yes” or “no,” with the number of “yes” items being the total score.

#### 3.2.2. School ACEs

The items were created by replacing the subject term of the five items of the ACEs questionnaire (physical abuse, verbal abuse, sexual abuse, emotional neglect, and witness of victims of violence) as follows: “Did a parent or other adult in the household …” was replaced to “Did a teacher or other adult in school (or preschool) ….” Additionally, two items related to bullying at school were added to the school ACE items: one item related to bullying victimization by classmates and the other item related to bullying victimization by upperclassmen. Responses are binary, “yes” or “no”, with the number of “yes” items being the total score.

#### 3.2.3. Depressive/anxiety symptoms

To assess depression and anxiety, the Patient Health Questionnaire-4 (PHQ-4) was used (49, 50). This scale consists of four items rated on a four-point Likert scale: two items extracted from the PHQ-9 (51) and two from the Generalized Anxiety Disorder-7 (GAD-7) (52). The total score was calculated, and the status of mental health problems was classified into four categories: normal (0–2), mild (3–5), moderate (6–8), and severe (9–12) (49). In this study, depressive/anxiety symptoms were dichotomized according to the level of severity as follows: moderate/high (6–12), or not (0–5).

#### 3.2.4. Hikikomori

We asked participants how often they go out, how long they have not been out including the reasons, and their current employment status. The “Guideline for the Assessment and Support of *Hikikomori*” defines *Hikikomori* as a phenomenological concept that refers to a state of avoidance of social participation (e.g., going to school, working, and socializing outside the home) as a result of various factors and remaining at home for 6 months or longer in principle (except for going out without socializing with others) (53). In accordance with this guideline, we defined a group of *Hikikomori* as follows: a person who remains at home for more than 6 months except for going out without socializing with others, who is not employed, a self-employed worker, a full-time homemaker, or a student and the reason for their current condition was not the result of illness, pregnancy, nursing care, effects of coronavirus disease 2019 (COVID-19) outbreak, or natural disasters.

#### 3.2.5. Background factors

Information on sex, age, nationality, education, number of family members living together, and economic conditions was collated as participants' background factors. The items were almost identical to those in the Cabinet Office's Survey (39). Item categories are shown in Table 1.

**Table 1:** Demographic characteristics of participants and prevalence of ACEs.

	**Total sample (*n* = 3,999)**
Sex: men, *n* (%)	1,976 (49.4)
Women, *n* (%)	1,977 (49.4)
Other, *n* (%)	46 (1.2)
Age, mean (SD)	27.2 (4.3)
Nationality: Japanese, *n* (%)	3,986 (99.7)
Other, *n* (%)	13 (0.3)
**Academic background:**	
Junior high school, *n* (%)	77 (1.9)
High school, *n* (%)	797 (19.9)
Vocational school, *n* (%)	411 (10.3)
Junior college, *n* (%)	253 (6.3)
College or graduate school, *n* (%)	2,409 (60.2)
Other, *n* (%)	52 (1.3)
Life circumstances (9-point scale), mean (SD)	4.7 (1.4)
Number of family members, mean (SD)	2.7 (1.4)
ACE total score, mean (SD)	0.76 (1.37)
Emotional abuse, *n* (%)	503 (12.6)
Physical abuse, *n* (%)	390 (9.8)
Sexual abuse, *n* (%)	162 (4.1)
Emotional neglect, *n* (%)	460 (11.5)
Physical neglect, *n* (%)	94 (2.4)
Divorce, *n* (%)	716 (17.9)
Mother treated violently, *n* (%)	210 (5.3)
Substance abuse, *n* (%)	177 (4.4)
Mental illness, *n* (%)	298 (7.5)
Incarcerated relative, *n* (%)	34 (0.9)
ACE > 1 point, *n* (%)	1,436 (35.9)
ACE > 4 point, *n* (%)	244 (6.1)
School ACE total score, mean (SD)	0.96 (1.18)
School ACE teacher-related score, mean (SD)	0.32 (0.75)
School ACE bullying-related score, mean (SD)	0.64 (0.71)
Emotional abuse, *n* (%)	297 (7.4)
Physical abuse, *n* (%)	149 (3.7)
Sexual abuse, *n* (%)	51 (1.3)
Emotional neglect, *n* (%)	577 (14.4)
Friends treated violently, *n* (%)	208 (5.2)
Bullying victimization by classmates, *n* (%)	1,924 (48.1)
Bullying victimization by senior students, *n* (%)	632 (15.8)
School ACE > 1 point, *n* (%)	2,202 (55.1)
PHQ-4 total score, mean (SD); median	2.65 (3.16); 2
PHQ-4 moderate/severe group, *n* (%)	653 (16.3)
Hikikomori group, *n* (%)	138 (3.5)

### 3.3. Statistical analysis

First, the total scores and prevalence rates of each item for ACEs and school ACEs were calculated. Next, the correlation between each ACEs item and school ACEs were examined through a correlation analysis. Separate logistic regression analyses were then performed for the depressive/anxiety symptoms and Hikikomori as outcomes, respectively. Potential confounding factors such as sex, age, education, living conditions, and number of family members were used to control the effects of ACEs and school ACEs. Since 99.7% of the participants was Japanese nationals, nationality was not included in the model. For the school ACEs score, we used the total score in Model 1 and divided it into teacher-related and bullying-related scores in Model 2.

In addition, we examined the impact of having ACEs and school ACEs on outcomes. Outcomes were moderate with higher levels of depressive/anxiety symptoms and the Hikikomori, and logistic regression analysis was used. Exposure was having both at least one ACE and at least one school ACE, or having both ≥4 ACEs and at least one school ACE.

## 4. Results

### 4.1. Prevalence of ACEs, school ACEs

Except for one participant who provided the same response to all questions, all participants (*n* = 3,999) had no missing data and were included in the analysis. Table 1 shows the background information of the sample and the prevalence of ACEs and school ACEs. The mean score of ACEs was 0.76 [standard deviation(SD) = 1.37], and 35.9% (*n* = 1,436) of the entire sample had at least one ACE. In addition, 6.1% (*n* = 244) had an ACEs score of ≥4. The mean score of the school ACEs was 0.96 (SD = 1.18), and 55.1% (*n* = 2,202) of the entire sample had at least one ACE. When dividing the school ACEs into teacher-related (five items) and bullying-related scores (two items), 20.5% (*n* = 819) of the entire sample had a score of ≥1 on the teacher-related items, and 50.5% (*n* = 2,020) had a score of 1 or more on the bullying items. Participants who showed moderate or higher levels of depressive or anxiety symptoms were 16.3% (*n* = 653), and participants assigned to the Hikikomori group were 3.5% (*n* = 138). Half (*n* = 69) of the Hikikomori group was assigned to the moderate or higher levels of depressive or anxiety group. This was significantly higher than the 15.1% (*n* = 584 out of *n* = 3,861) in the non-Hikikomori group (χ^2^ = 118.6, p < 0.001).

Table 2 shows the correlations between each item of ACEs and school ACEs. Within the 10 items of ACEs, strong correlations were observed between emotional abuse and physical abuse, as well as between emotional neglect and physical abuse. Within the seven items of school ACEs, moderate correlations were observed between some items; however, no strong correlations were found. The correlation coefficient between the total score of ACEs and school ACEs was 0.41, indicating a moderate correlation.

**Table 2:** Correlations among each item of ACEs and school ACEs.

	**ACEs**											**School ACEs**							
												**Teacher-related items**					**Bullying-related items**		
	**1**	**2**	**3**	**4**	**5**	**6**	**7**	**8**	**9**	**10**	**Total**	**11**	**12**	**13**	**14**	**15**	**16**	**17**	**Total**
1. Emotional abuse	1																		
2. Physical abuse	0.54	1																	
3. Sexual abuse	0.18	0.16	1																
4. Emotional neglect	0.50	0.35	0.17	1															
5. Physical neglect	0.30	0.28	0.19	0.30	1														
6. Divorce	0.17	0.17	0.10	0.12	0.11	1													
7. Mother treated violently	0.38	0.50	0.13	0.23	0.27	0.15	1												
8. Substance abuse	0.26	0.20	0.19	0.22	0.22	0.16	0.22	1											
9. Mental illness	0.22	0.20	0.13	0.20	0.18	0.13	0.21	0.22	1										
10. Incarcerated relative	0.11	0.13	0.16	0.07	0.18	0.09	0.10	0.18	0.13	1									
Total score of ACEs	0.73	0.69	0.39	0.63	0.49	0.49	0.59	0.49	0.48	0.27	1								
11. Emotional abuse in school	0.26	0.23	0.13	0.22	0.22	0.05	0.20	0.09	0.13	0.12	0.30	1							
12. Physical abuse in school	0.14	0.21	0.10	0.12	0.13	0.04	0.18	0.08	0.08	0.14	0.21	0.33	1						
13. Sexual abuse in school	0.11	0.11	0.26	0.06	0.20	0.06	0.13	0.15	0.09	0.23	0.22	0.20	0.20	1					
14. Emotional neglect in school	0.25	0.16	0.14	0.31	0.15	0.08	0.13	0.11	0.12	0.06	0.29	0.37	0.18	0.13	1				
15. Friends treated violently in school	0.18	0.20	0.09	0.18	0.19	0.05	0.17	0.10	0.12	0.13	0.25	0.37	0.33	0.20	0.24	1			
16. Bullying victimization by classmates	0.20	0.18	0.08	0.18	0.10	0.06	0.11	0.09	0.12	0.03	0.22	0.19	0.09	0.03	0.20	0.18	1		
17. Bullying victimization by senior students	0.15	0.14	0.08	0.15	0.11	0.04	0.10	0.09	0.11	0.08	0.19	0.24	0.18	0.05	0.17	0.20	0.32	1	
Total score of school ACEs	0.33	0.30	0.18	0.32	0.24	0.10	0.24	0.16	0.19	0.15	0.41	0.63	0.46	0.28	0.60	0.55	0.68	0.62	1

Light gray indicates a moderate correlation, while dark gray indicates strong correlation.

### 4.2. Association with mental health and Hikikomori

Table 3 shows the association between moderate or higher levels of depressive/anxiety symptoms and ACEs and school ACE scores. In Model 1, a significant association was found between ACE scores and depressive/anxiety symptoms, with a 24% increased risk of depressive/anxiety for every one-point increase in the ACEs score. School ACE scores were also significantly associated with depressive/anxiety symptoms, with a 44% increased risk for every one-point increase in the school ACEs score.

**Table 3:** Effects of ACE and school ACE on depression/anxiety.

	**Model 1**	**Model 2**
	**OR (95% CI)**	**OR (95% CI)**
ACE total score	1.24 (1.17, 1.32)^*^	1.25 (1.17, 1.33)^*^
School ACE total score	1.44 (1.39, 1.55)^*^	
School ACE teacher-related score		1.33 (1.19, 1.49)^*^
School ACE bullying-related score		1.60 (1.40, 1.83)^*^
Sex (female)	1.07 (0.89, 1.29)	1.07 (0.89, 1.29)
Age	0.97 (0.95, 0.99)^*^	0.97 (0.95, 0.99)^*^
Academic background	0.94 (0.88, 1.01)	0.94 (0.88, 1.01)
Life circumstances	0.68 (0.64, 0.73)^*^	0.68 (0.64, 0.73)^*^
Number of family members	1.03 (0.96, 1.10)	1.03 (0.96, 1.10)

OR, odds ratio; CI, confidence interval; ^*^p < 0.05.

The total score of the school ACEs was used in Model 1, and it was divided into teacher-related and bullying-related scores in Model 2.

In Model 2, the risk of depressive/anxiety symptoms was significantly increased in both ACE scores and school ACE scores for teacher-related and bullying-related factors. The potential confounding factors showed that age had a significant effect, with a decreased risk of depressive/anxiety symptoms with increasing age. Additionally, more favorable life circumstances were associated with a decreased risk of depressive/anxiety symptoms.

The association between Hikikomori and ACEs and school ACE scores is shown in Table 4. In Model 1, ACE scores did not show a significant association with Hikikomori, except for school ACE scores: a 29% increased risk of Hikikomori for every one-point increase in school ACE scores. In Model 2, both school ACE scores for teacher-related and bullying-related factors showed a significant association with Hikikomori; the rates for the increased risk were 23 and 37%, respectively. Additionally, having more family members increased the risk of Hikikomori. However, a higher academic background and more favorable life circumstances were associated with a decreased risk of Hikikomori.

**Table 4:** Effects of ACE and school ACE on Hikikomori.

	**Model 1**	**Model 2**
	**OR (95% CI)**	**OR (95% CI)**
ACE total score	1.01 (0.89, 1.13)	1.01 (0.90, 1.14)
School ACE total score	1.29 (1.13, 1.47)^*^	
School ACE teacher-related score		1.23 (1.01, 1.51)^*^
School ACE bullying-related score		1.37 (1.06, 1.78)^*^
Sex (female)	0.84 (0.58, 1.21)	0.83 (0.58, 1.21)
Age	0.99 (0.95, 1.03)	0.99 (0.95, 1.03)
Academic background	0.65 (0.57, 0.74)	0.65 (0.57, 0.74)
Life circumstances	0.65 (0.58, 0.74)	0.65 (0.58, 0.74)
Number of family members	1.14 (1.01, 1.30)^*^	1.14 (1.01, 1.29)^*^

OR, odds ratio; CI, confidence interval; ^*^p < 0.05.

The total score of the school ACEs was used in Model 1, and it was divided into teacher-related and bullying-related scores in Model 2.

Of the 1,436 who had at least one ACE, 1,027 (71.5%) had at least one school ACE. Of the 244 who had ≥4 ACEs, 211 (86.5%) had at least one school ACE. With both at least one ACE and at least one school ACE, the odds ratio for depressive/anxiety symptoms was 3.59 [95% confidence interval (CI): 3.00, 4.28; *p* < 0.001] and the odds ratio for Hikikomori was 2.48 (95% CI: 1.74, 3.53; *p* < 0.001) compared with those who did not have both. With both ≥4 ACEs and at least one school ACE, the odds ratio for depressive/anxiety symptoms was 4.38 (95% CI: 3.28, 5.86; *p* < 0.001) and the odds ratio for Hikikomori was 2.11 (95% CI: 1.22, 3.65; *p* = 0.01). The impact on depressive/anxiety symptoms is particularly pronounced when both adversity experiences are cumulative.

## 5. Discussion

The present study is one of the first few studies to integrate adverse school experiences within the ACEs framework (as school ACEs), and examine their prevalence, as well as their impact on mental health and adjustment in adulthood. Importantly, the prevalence of school ACEs was much higher than traditional ACEs, suggesting that many people have more adverse childhood experiences in school than at home. The findings of this study also reveal that school ACEs are linked to both declining mental health in adulthood and Hikikomori. Regarding the latter, no association was found in traditional ACEs such as abuse at home or family dysfunction, suggesting that school ACEs play a crucial role as contributing factors.

As responsible members of society, recognizing the significance of schools inflicting harm on children and the subsequent negative effects that persist into adulthood is necessary.

### 5.1. Descriptive statistics

The prevalence of school ACEs is higher in Japan than the previously reported prevalence rates of extra-familial (including ACEs at school) ACEs (22). Several factors may contribute to this difference. First, the previous study sample was limited to university students, whereas our study included a broader range of participants. Second, while the previous study examined physical violence from teachers and negative perceptions as extra-familial ACEs, our proposed concept of school ACEs encompasses additional aspects such as emotional neglect and witnessing friends being treated violently by teachers. Notably, emotional neglect has the highest prevalence within the school ACEs teacher-related items. Both emotional neglect and witnessing violence toward friends indicate an overall unsafe school environment for children, even if they have not personally experienced direct harm. These findings suggest that children can be emotionally harmed by the actions of teachers, regardless of whether they have experienced direct victimization.

The rate of bullying victimization by classmates was 48.1% and that by upper classmen was 15.8%. This is higher than the bullying victimization rate of 33.6% in Osuka et al.'s (54) survey of Japanese elementary and junior high school students. However, this can be attributed to the fact that Osuka et al.'s survey directly targeted elementary and junior high school students and only covered bullying victimization in the 3 months prior to the survey, while this survey targeted bullying victimization during the entire period before the age of 18 in adulthood.

In this survey, the prevalence of Hikikomori was 3.5%. Although simple comparisons cannot be made because of the different ages targeted, considering that the prevalence of Hikikomori reported by the Cabinet Office of Japan (39) was 2.05%, the result was 1.7-fold higher. However, another survey conducted in Tokyo reported the prevalence of Hikikomori as 4.39% (55). The prevalence of this study was closer to the latter.

The proportion of those who scored at least one ACE in this survey was 35.9%, which is similar to the results of previous studies on Asian populations, as well as previous studies in Japan (30–40%). This indicates that this survey, despite being conducted online, accurately captured the situation of ACEs in Japanese people.

The strong correlations found for some of the ACE items may indicate that multiple adverse experiences tend to overlap because the perpetrator is the same person, while the only moderate correlations among the school ACE items may have been related to the fact that teachers and classmates change every year. Nevertheless, the moderate correlations still suggest that multiple adversity experiences are more likely to be cumulative within a school, and the impact of cumulative experiences needs to be further examined.

### 5.2. Association with mental health

Our findings provide evidence supporting the association between ACEs and mental health problems in adulthood, which is consistent with previous research (1, 3, 4, 12, 46). The results also support the hypothesis of our study, whereby school ACEs are related to mental health problems in adulthood. Previous research has emphasized the significance of adverse experiences outside the home (22), indicating the need to expand the concept of ACEs (20). Moreover, there is supporting evidence linking bullying victimization and mental health problems in adulthood (24, 56).

Additionally, ACEs can also affect academic performance (33) and behavioral problems (57, 58), rendering children with ACEs more susceptible to teacher reprimands and bullying. Thus, considering the possibility that ACEs may play a role in the association between school-related ACEs and mental health deterioration in adulthood is necessary. It should be emphasized that regardless of the reasons, it is unacceptable for schools, whose responsibility is to safeguard children's wellbeing and enable their development, to inflict hurt and contribute to mental health problems. Therefore, a reevaluation of the behavior of teachers toward their students within school settings is therefore warranted.

### 5.3. Association with Hikikomori

The novel and significant finding of this study is that school ACEs have a strong association with Hikikomori, whereas ACEs within the family do not. This has not been previously reported in the literature. It highlights how schools, which should protect children's development, can actually cause harm and profoundly impact their social adaptation.

Individuals with Hikikomori have difficulty with social participation (36) and tend to feel safe at home (38). Thus, it is plausible that experiences at school (school ACEs), where students interact with society, have a substantial impact compared to ACEs within the family. Kato et al. proposed a model for the development of Hikikomori, in which the school environment triggers bullying victimization or scolding by teachers, leading to avoidance behavior (37). The influence of the family environment is reflected in personality traits and individual characteristics, but it is not considered a causative factor for Hikikomori. In this survey, conventional ACEs were not associated with Hikikomori, while school ACEs were, supporting this model.

The findings of this study reveal that experiences of bullying victimization and mistreatment by teachers increase the risk of mental health problems and further contribute to the risk of Hikikomori, thereby hampering social participation. These experiences have significant societal implications; it has the potential to add to societal burden. Kato et al. have previously highlighted the importance of addressing bullying victimization and mistreatment by teachers (37), which are now specifically identified as school ACEs. Further research and interventions are warranted to address these issues. Recent research suggests that mistreatment within schools may be a significant factor contributing to school refusal in Japan (59). A survey conducted by the Cabinet Office of Japan in 2020 listed school refusal as a prominent cause of Hikikomori (60). These findings, together with the results of this study, imply that school ACEs are associated with Hikikomori through the intermediary factor of school refusal.

In the current trend of ACE research, efforts are being exerted to identify protective factors that can mitigate the risks associated with ACEs in adulthood. For example, the framework of health outcomes of positive experiences was introduced by Sege and Harper Browne (61), which suggests that positive childhood experiences can help alleviate the impact of ACEs. Bethell et al. (62) identified positive childhood experiences (PCEs) as protective factors that work in adulthood, such as safe family environments and friendships, and seven items were established. They demonstrated that these protective factors work even for those with high ACE scores. Robles et al. (63) identified three protective factors related to the family and four related to the community that counteract the negative impact of ACEs on academic performance in school. Therefore, it is imperative for us to address school ACEs while also identifying school PCEs. Such research holds the potential to ultimately prevent societal issues such as school refusal, youth unemployment, and poverty.

## 6. Limitations

This study has several limitations. First, this study was conducted on a web-based survey, recruiting a total of 4,000 participants of different sexes and age groups. As the survey was conducted among survey collaborators owned by private companies, it is likely that a bias exists toward individuals who are “willing to participate in such surveys” and are “internet-friendly”. However, it can be concluded that this study reflects the overall situation to a certain extent considering that web-based surveys have become prevalent and the descriptive statistics reveal no significant difference from previous survey results. Second, this survey focuses on ACEs before the age of 18, including school ACEs, which may be affected by recall bias. However, given the lack of consistently strong correlations between ACEs and school ACEs, it suggests that participants are likely differentiating and providing distinct responses for ACEs and school ACEs. Third, this survey did not measure neurodevelopmental conditions, such as autism spectrum disorder or attention deficit hyperactivity disorder, which are associated with a higher risk of bullying victimization (64, 65) and ACEs (66). Furthermore, while Hikikomori has been associated with schizophrenia, social anxiety disorder, personality disorders, and depression (36), this survey did not collect information on such mental illnesses. Therefore, the influence of neurodevelopmental and psychiatric conditions on the results of this survey remains unclear. Fourth, we included two types of bullying-related items in school ACEs: one item related to bullying victimization by classmates and the item related to bullying victimization by upperclassmen. However, these may not be considered separate experiences. Rather, it would have been more appropriate to ask about the types of bullying, such as physical, psychological, and sexual bullying, in conjunction with the teacher-related items. Future studies will need to further examine the school ACE items. Finally, this survey was conducted in 2021 during the COVID-19 pandemic, which had a significant impact on social and economic life. Therefore, COVID-19 might have affected the mental health and social withdrawal outcomes of this survey, and this confounding factor should be highlighted.

## 7. Conclusions

This study provides a new concept of school ACEs, which is an extension of the conventional ACEs framework. The results indicate that school ACEs may have a more serious impact on social participation than conventional ACEs. This highlights the increasing importance of schools, as places for children's social participation, in providing a safe and secure environment to ensure children's healthy development and promotion of their wellbeing.

By situating negative experiences in schools within the context of ACE research and comparing them with traditional ACEs, this study has elucidated their severity and nature. Positive experiences in schools, such as active engagement with school, school climate, and achievements, are known to have a positive impact on the future but have not been integrated into PCEs research.

Through the examination of school experiences within the framework of ACEs and PCEs research, the role of schools in the future society becomes apparent. This insight can help clarify what we should or should not provide to children. Given the substantial influence of schools on children's lives, it is essential to further explore the complexity of school ACEs while simultaneously uncovering the realm of school PCEs. Consequently, comprehensive research on school ACEs, coupled with an in-depth investigation of school PCEs in the future, is imperative.

## Data availability statement

The raw data supporting the conclusions of this article will be made available by the authors, without undue reservation.

## Ethics statement

The studies involving humans were approved by the Hirosaki University Ethics 205 Committee. The studies were conducted in accordance with the local legislation and institutional requirements. The participants provided their written informed consent to participate in this study.

## Author contributions

MW: Conceptualization, Data curation, Funding acquisition, Investigation, Project administration, Writing—original draft, Writing—review & editing. TN: Data curation, Formal analysis, Investigation, Methodology, Validation, Writing—review & editing. YO: Writing—review & editing. NT: Writing—review & editing. MT: Writing—review & editing. MA: Writing—review & editing. TS: Writing—review & editing. TK: Writing—review & editing.
