# Supplementary material for: Adverse childhood experiences: impacts on adult mental health and social withdrawal
Source: Front Public Health. 2023 Oct 26;11:1277766. doi: 10.3389/fpubh.2023.1277766 (PMC10639139; doi:10.3389/fpubh.2023.1277766)
Supplement: Supplementary file 1 [file Data_Sheet_1.PDF]

School Adverse Childhood Experiences (School ACEs)

We would like to ask you questions about your time in kindergarten, elementary school, middle school, and high school.

1. Did an Adults at schools (including kindergartens and nursery schools; the same applies hereinafter), such as teachers often or very often…
   Swear at you, insult you, put you down, or humiliate you? or
   Act in a way that made you afraid that you might be physically hurt?
   ◯ Yes ◯ No

2. Did an Adults at schools (including kindergartens and nursery schools; the same applies hereinafter), such as teachers often or very often… Push, grab, slap, or throw something at you? or Ever hit you so hard that you had marks or were injured?
   ◯ Yes ◯ No

3. Did an Adults at schools (including kindergartens and nursery schools; the same applies hereinafter), such as teachers ever…
   Touch or fondle you or have you touch their body in a sexual way? or
   Attempt or actually have oral or anal intercourse with you?
   ◯ Yes ◯ No

4. Did you often or very often feel that …
   No one in your school treated you or thought you were important or special? or
   An adults at schools didn't look out for each other, feel close to each other, or support each other? ◯ ◯ Yes ◯ No

5. Was your friends (classmates, seniors):
   Often or very often pushed, grabbed, slapped, or had something thrown at him/her? or
   Sometimes, often, or very often kicked, bitten, hit with a fist, or hit with something hard? or
   Ever repeatedly hit over at least a few minutes or threatened with a gun or knife?
   ◯ YeS ◯ No

6. Have you ever transferred or dropped out of school?
   ◯ Yes ◯ No

7. Have you ever been teased, yelled at, punched, beaten, left out, had things taken from you, or made to do something you didn't like by a friend (classmate)?
   ◯ Yes ◯ No

8. Have you ever been teased, yelled at, punched, beaten, left out of your group, had things taken from you, or made to do something you didn't like by your seniors?

   ◯ Yes ◯ No
